# Galacto-Oligosaccharide/Polidextrose Enriched Formula Protects against Respiratory Infections in Infants at High Risk of Atopy: A Randomized Clinical Trial

**DOI:** 10.3390/nu10030286

**Published:** 2018-03-01

**Authors:** Giusy Ranucci, Vittoria Buccigrossi, Eleonora Borgia, Daniela Piacentini, Federica Visentin, Luigi Cantarutti, Paola Baiardi, Mariagrazia Felisi, Maria Immacolata Spagnuolo, Stefania Zanconato, Eugenio Baraldi, Carlo Giaquinto, Alfredo Guarino

**Affiliations:** 1 Unit of Pediatrics, Department of Translational Medical Science, University Federico II, Via Pansini 5, 80131 Naples, Italy; buccigro@unina.it (V.B.); mispagnu@unina.it (M.I.S.); alfguari@unina.it (A.G.); 2 Bambino Gesù Children’s Hospital, Division of Metabolism, Piazza di Sant’Onofrio, 4, 00165 Rome, Italy; 3 Department of Women’s and Children’s Health, University of Padua, Via Giustiniani 3, 35128 Padua, Italy; eugenio.baraldi@unipd.it (E.B.); daniela.piacentini@hotmail.it (D.P.); fede82.visentin@gmail.com (F.V.); zanconato@pediatria.unipd.it (S.Z.); eleonborgia@gmail.com (E.B.); carlo.giaquinto@unipd.it (C.G.); 4 Pedianet Network, 35128 Padua, Italy; l.cantarutti@sosepe.com; 5 Istituti Clinici Scientifici Maugeri SpA Benefit Corporation, Via S. Severino Boezio, 28, 27100 Pavia, Italy; paola.baiardi@icsmaugeri.it; 6 Consorzio per Valutazioni Biologiche e Farmacologiche, Via Luigi Porta, 14, 27100 Pavia, Italy; mfelisi@cvbf.net

**Keywords:** nutrition, microbiome, general practice, atopic dermatitis, respiratory infections, acute gastroenteritis, prebiotics

## Abstract

Background: Early nutrition affects the risk of atopy and infections through modifications of intestinal microbiota. The Prebiotics in the Prevention of Atopy (PIPA) study was a 24-month randomised, double-blind, placebo-controlled trial. It aimed to evaluate the effects of a galacto-oligosaccharide/polydextrose (GOS/PDX)-formula (PF) on atopic dermatitis (AD) and common infections in infants who were born to atopic parents and to investigate the relationship among early nutrition, gut microbiota and clinical outcomes. Methods: A total of 201 and 199 infants were randomized to receive a PF and standard formula (SF), respectively; 140 infants remained on exclusive breastfeeding (BF). Results: The cumulative incidence of AD and its intensity and duration were not statistically different among the three groups. The number of infants with at least one episode of respiratory infection (RI) and the mean number of episodes until 48 weeks of age were significantly lower in the PF group than in the SF group. The number of patients with recurrent RIs and incidence of wheezing lower RIs until 96 weeks were lower in the PF group than the SF group, but similar to the BF group. Bifidobacteria and Clostridium cluster I colonization increased over time in the PF group but decreased in the SF and BF groups. Bifidobacteria had a protective role in RIs, whereas Clostridium cluster I was associated with atopy protection. Conclusion: The early administration of PF protects against RIs and mediates a species-specific modulation of the intestinal microbiota. Trial registration: clinicaltrial.gov Identifier: NCT02116452.

## 1. Introduction

Human milk contains more than 200 nondigestible milk human oligosaccharides (HMO), whereas cow’s milk contains virtually no oligosaccharides, which explains the greater abundance of intestinal bifidobacteria observed in infants who were fed human milk compared with standard formula (SF) [1,2,3]. Nutritional regimen greatly shapes intestinal microbiota composition, with impact on future human health and specific clinical outcomes [4]. This may be particularly important in early life when initial microbiological colonization drives immune imprinting. Immune development affects the subsequent risk of allergy and of infections; at the same time atopy correlates with increased risk of wheezing and asthma, although data are controversial [5].

Selected prebiotics, such as galactooligosaccharides (GOS) and inulin, have a bifidogenic effect when added to milk formula [4]. Most effects have been observed with two chemical groups, namely inulin-type fructans and GOS. Those effects have clinical implications and preliminary evidence shows that addition of prebiotics to milk formula results in better stool pattern, reduces the risk of gastroenteritis and respiratory infections (RIs), reduces the incidence of atopic eczema and promotes mineral absorption [4]. Further research is necessary before routine use of prebiotics can be recommended for the prevention of allergy in formula-fed infants. Whether the use of prebiotics should be restricted to infants at high risk of allergy or may have an effect in low-risk populations and whether it may have an effect on other allergic diseases including asthma are unclear [6]. Furthermore, the cause-effect relationship with intestinal microbiota and the true clinical importance of such effects are not yet fully elucidated. The Committee on Nutrition of the European Society for Pediatric Gastroenterology, Hepatology and Nutrition stated that “there is no sufficient evidence of clinical benefits to recommend the addition of prebiotics to infant foods” [6].

The main limitations of studies on prebiotics include small sample size, difficulties of having a proper control population of breastfed infants, a lack of stringent inclusion and exclusion criteria (such as the specific risk of study populations), a lack of standardised endpoints, and the absence of parallel clinical and microbiological data [7,8,9,10,11].

GOS selectively enhances the growth of Bifidobacteriaceae and Lactobacillaceae, and increases the colonic production of short-chain fatty acids that decrease luminal pH, which may reduce the growth of enteropathogens [4]. Infant formula containing the prebiotic mixture GOS/fructooligosaccharide (FOS) compared with a standard infant formula, was associated with a significant reduction in the incidence of gastroenteritis, but not of upper respiratory tract infections although the number of children with multiple antibiotic courses per year was lower in children receiving prebiotics [8]. One randomized controlled trial (RCT) found a similar number of episodes of diarrhoea and fewer episodes of physician-diagnosed respiratory tract infections, fever episodes and fewer antibiotic prescription in the group of infants fed extensively hydrolysed whey formula supplemented either with 0.8 g GOS/FOS or with maltodextrin as placebo [9]. Two RCTs investigated the role of prebiotic supplementation of infant formula for the prevention of allergic disease and food hypersensitivity [10,11]. One of the included RCT investigated the effect of GOS/FOS on the cumulative incidence of atopic dermatitis during the first six months of life in infants at risk for allergy, and showed a reduction of atopic dermatitis (AD) in the first year of life [10]. However there are conflicting data about the protective effect beyond the first year of life [10,12]. In a large multicentre, randomised, double-blind and placebo-controlled trial in 365 healthy term infants enrolled before eight weeks of age, the administration of GOS-containing infant formula without other HMOs, despite producing a definite prebiotic effect, did not change the incidence of infections or allergic manifestations in the first year of life [13]. The relation between respiratory illnesses in early life and the development of asthma and atopy in childhood is incompletely understood. However no study evaluated whether prebiotics modify the history of common infections in a population with high risk of atopy.

Polydextrose (PDX) is a peculiar HMO whose effects in vivo and in vitro are less investigated than GOS. In contrast to other prebiotics with smaller molecular weights, PDX is fermented and remains available as a carbon source for microbiota with a sustained production of short-chain fatty acids (acetate, propionate, butyrate) [14]. PDX-containing infant formula increases ileal lactobacilli and propionic and lactic acid concentrations, and decreases pH with associated changes in ileal cytokine expression [14].GOS and PDX have generally-recognised-as-safe (GRAS) status and is often added to commercial infant formula [15].

The aim of the Prebiotics in Prevention of Atopy (PIPA) study was to evaluate whether the addition of GOS/PDX to infant formula administered to young infants at risk of atopy prevents AD and reduces the risk of respiratory infections (RIs) and acute diarrhea in the first two years of life. A microbiological substudy was conducted in parallel in order to analyze the composition of intestinal microbiota in relation to nutritional intervention. We also included a control group of breastfed infants as breastfeeding (BF) is the gold standard of nutrition in infants until six months.

## 2. Materials and Methods

### 2.1. Clinical Trials.Gov Identifier

NCT02116452. The study protocol was approved by the ethics committee of the University of Padua. This double blind, randomized controlled trial investigated the effects of a GOS/PDX supplemented formula compared with a standard formula (SF) in preventing and modifying the history of AD in the first two years of life in infants at risk of atopy. Secondary endpoints included incidence of common infections (RIs and acute diarrhea in infants at risk of atopy).

### 2.2. Patient Selection Criteria

Infants who met all of the following criteria were eligible in the study: (1) gestational age > 37 and <42 weeks; (2) birth weight > 2500 g; (3) at risk of atopy (with at least one parent with physician-diagnosed asthma, allergic rhinoconjunctivitis, AD, allergic urticaria and food allergy) [16]; and (4) informed consent. Infants were screened for risk of atopy at delivery using a validated questionnaire [11]. Exclusion criteria were as follows: congenital immunodeficiency, severe congenital disorders or malformations, infants born to mothers with diabetes, long-term intake (>7 consecutive days) of probiotics or prebiotics, and intake of ≥50 mL of formula different from that included in the study for at least a week.

### 2.3. Randomisation

**Figure 1:**
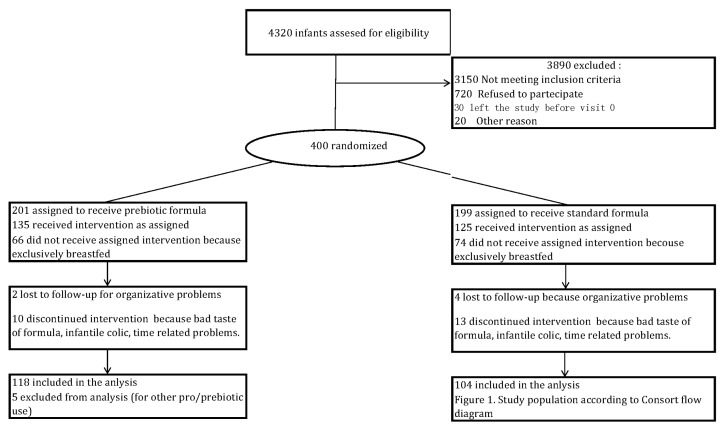
Study population according to CONSORT flow diagram.

At birth, infants were randomised to either the SF (control group) or study formula (treatment arm) to be taken until 48 weeks of life. Only infants who had taken the formula were included in the analysis. Randomised infants who ended up being exclusively breastfed and did not receive artificial milk formula until at least six months of age and continued BF constituted the parallel comparison group. Figure 1 summarises the study population according to the CONSORT (CONsolidated Standards of Reporting Trials) flow diagram.

### 2.4. Study Product

At birth, patients were randomized to receive 50/50 GOS/PDX formula (prebiotic formula [PF]) or SF until 48 weeks of life. The two formulas were identical except for the addition of a mixture of 4 g/L of GOS/PDX in a ratio of 50/50. The entire amount of formula needed for each child was provided by Mead Johnson Nutrition (Evansville, IN, USA) in a similar package to keep the randomized group blind both to the physicians and parents. Patients who were exclusively breastfed up to 24 weeks of life constituted the parallel comparison group of breastfed infants.

### 2.5. Patient Follow-up

At enrolment, infants at 24 (visit 1), 36 (visit 2), 48 (visit 3) and 96 (visit 4) weeks of age were evaluated by specifically trained physicians in the pneumology and allergology ambulatory of the University of Padua. All of the mothers were provided with a follow-up patient diary. Parental compliance with feeding recommendations was assessed based on diary entries in addition to structured interviews on health problems reported since the last medical visit.

### 2.6. Outcomes

Primary endpoints comprised the incidence and severity of AD at 36 weeks and 48 weeks of life. The AD was diagnosed according to the criteria described by Halken et al. [17] and Muraro et al. [18]. Only patients with a definitive physician diagnosis were included as cases in the statistical analysis.

Primary outcome parameters to define AD included the cumulative number of patients with at least one episode of AD at 36 weeks and 48 weeks of life, and the severity of AD at 36 and 48 weeks of life, as graded using the SCORAD (SCORing Atopic Dermatitis) score. All patients with atopic dermatitis was evaluated by food allergy prick tests to define food sensitization. Secondary endpoints to analyze AD included the cumulative number of patients with at least one episode of AD at 96 weeks of life; the severity of AD at 96 weeks of life graded using the SCORAD score; the mean number of episodes of AD at 36, 48 and 96 weeks of life; the total duration of AD (in days) at 48 weeks and 96 weeks of life; the use of drugs to treat AD at 48 weeks and 96 weeks of life (quantity and type). Secondary endpoints were also comprised of parameters to analyze the occurrence of (RIs). RIs were defined as any infection of three upper and lower respiratory tract diagnosed by a physician [19]. Recurrent RIs (RRIs) will be defined as more than 3 episodes/year of RIs requiring medical attention [5]. Parameters considered to estimate RIs included the cumulative number of infants with at least one episode of RI at 48 weeks and 96 weeks of life, the cumulative number of infants with RRIs at 96 weeks of life, the mean number of RI episodes at 48 and 96 weeks of life, the mean duration of RIs, the cumulative number of infants with wheezing lower RIs at 48 weeks and 96 weeks, number of patients with at least one antibiotic prescription, the number of parents who lost workdays within the first 96 weeks of life of infants, as well as the number of days lost at work by parents for children infections, and the number of infants with at least one episode of acute diarrhea at 48 weeks and 96 weeks of life. Acute gastroenteritis (AG) is defined as a reduction in the consistency of stools (loose or liquid) and/or an increase in the frequency of evacuations (typically three or more/24 h), with or without fever, vomiting, dehydration, or compromised general status during less than 7 days. Clinical outcomes were reported by parents in the weekly diary and confirmed by primary care physicians (family paediatricians or FPs). FPs belonged to the established Italian network Pedianet, covering a well defined number of patients in the Veneto Region of Italy.

### 2.7. Microbiological and Faecal Markers

A substudy was conducted to evaluate gut microbiota composition in 147 infants (45%). Stool samples were collected at baseline (before starting the nutritional intervention, with age ranging from one to six months) and at 9–12 months of age. Gut microbiota composition was investigated using fluorescence in situ hybridisation. The bacterial species analyzed were Bifidobacteria, Clostridium cluster I, Clostridium cluster IX, Firmicutes, Bacteroides and Faecalibacterium prausnitzii. The species were selected based on the possible relationships with clinical outcomes reported in previous papers [20,21].

Microbiological data were obtained at baseline (before nutritional shift) and at 9–12 months of age corresponding to a minimum period of three months of specific nutritional regimen. The loads of microbial species in the post-intervention period compared with baseline were measured to investigate their possible relationship with the type of feeding.

Fecal samples were fixed in Carnoy’s solution and embedded in paraffin, which was cut longitudinally into four sections, placed on Super- Frost slides (Thermo Scientific, Monza MB, Italy), and incubated with hybridization solution (20 mM Tris-HCl, 0.9 M NaCl, 0.1% SDS, and 1% formamide, pH 7.4) with EUB mix positive control oligonucleotide probes (100 ng of EUB I, EUBII, and EUBIII). For specific bacteria groups, the slides were incubated with hybridisation buffer with 25 ng of their respective fluorescent in situ hybridization (FISH) probes for 45 min at 50 °C and visualised using a Nikon 80i Eclipse epifluorescence microscope with a Nikon DS-U2 color camera and NIS-Elements imaging software (Nikon, Tokyo, Japan). Bacteria were quantified by count. The oligonucleotide probes used in this study were synthesized by MWG Eurofins (MWG Operon Ebersberg, Ebersberg, Germany). All of the samples were analysed with the Eub338 mix probe conjugated with fluorescein isothiocyanate (FITC, green signal) at the end (positive control that reacts with all bacteria), and a species-specific probe was conjugated with a single fluorescent carbocyanine molecule (Cy3, red signal).

### 2.8. Statistical Methods

Sample size calculation was calculated a priori. It was based on the difference in AD incidence between GOS/PDX and SF at 48 weeks. Assuming that the proportions of infants expected to develop AD in the PF and SF groups being are equal to 30% and 50%, respectively, considering type I and II errors of 0.05 and 0.20 and a dropout rate of 10%, the estimated sample size was 120 infants per randomized arm (240 in total for the formula groups). As a result the power of the study was calculated to emphasize a difference of 20%.

The analysis and results reported in this paper reflect the final study design and are based on the per protocol (PP) population including all children completing the study. Intention-to-treat analysis (ITT) confirmed the data trend. Descriptive statistics were reported for all of the recorded variables; frequencies and percentages for qualitative variables, medians and interquartile ranges for ordinal variables and means and standard deviations for quantitative variables were applied. The chi-square test and one-way analysis of variance were conducted to determine the differences among the three groups (PF, SF and BF) in the qualitative and quantitative variables, respectively. In case of significant or nearly significant overall tests (*p* < 0.10), post-hoc tests were performed to identify pairwise differences. Bonferroni correction was applied for multiplicity.

## 3. Results

### 3.1. Patients

A total of 4320 infants born in Padua were screened at birth from September 2011 to December 2014 for eligibility, with a questionnaire to evaluate the risk of allergy. Four hundred eligible patients were randomised: 201 infants received a prebiotic (GOS/PDX)-enriched formula (PF) and 199 infants received an SF until 48 weeks of life if BF was insufficient. Between these two groups, 140 infants remained on exclusive breastfeeding until six months of ageand were included in the parallel comparison group. A total of 345 patients completed the randomised study, and were included in the PP population: 118, PF; 104, SF; and 123, BF. Fifty-five were lost to follow-up and excluded from the PP analysis. The reasons for exclusion from the PP population were missing endpoint [22], use of other pre/probiotics [9], formula refused or change, infantile colics, and noncompliance with standard procedures [22]. Baseline population features were similar in the PP and ITT populations.

The prevalence of caesarean delivery in the studied population was 31.6% with no difference between the two randomised formula groups. Comparison of the two groups of infants receiving formula and the BF group did not show any difference. The three groups were comparable for all the baseline features, except the father’s education (based on the last level of education, as lower secondary, upper secondary and degree). Parent’s education level was used as an indicator of social status. Baseline characteristics of the three study groups are summarised in Table 1.

### 3.2. Clinical Outcomes

#### 3.2.1. Atopic Dermatitis

**Figure 2:**
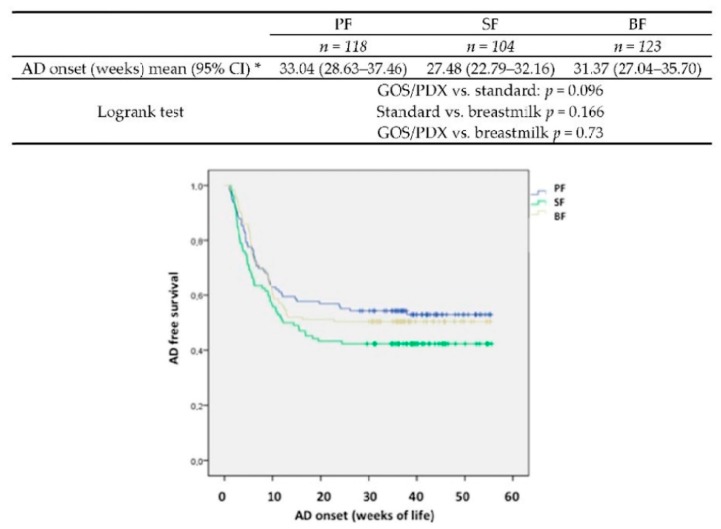
Atopic dermatitis onset according to type of feeding. AD: atopic dermatitis; PF: prebiotic formula; SF: stan.

Overall, since two years old, 177 (51%) infants have manifestations of AD (52% of formula-fed (FF) and 50% of BF infants). The number of children who developed AD until 96 weeks was significantly higher in children born by caesarean than natural route (*p*: 0.021). The cumulative number of infants with at least one episode of AD was not statistically different between the PF and SF infants at 36 weeks, 48 weeks and 96 weeks. No advantage has been showed for PF group according to all of the AD analysed variables (Table 2). A Kaplan-Meyer curve showed that the onset of AD occurrence was more delayed in PF than in SF fed infants (*p*: 0.096) (Figure 2). The SCORAD scores in all of the groups were progressively reduced in growing infants with the same pattern in the three groups. 

Accordingly, in the Cox model (Table 3), the rate of AD was reduced by 35% in PF compared with SF (*p*: 0.09) and by 38% in BF compared with SF (*p*: 0.001). Among the considered covariates, “age at introduction of solid food” had a significant effect on this outcome, with a higher risk of AD in children weaned after six months of life (*p*: 0.03). The number needed to treat to prevent one case of AD, calculated as 100 divided by the difference in the percentage of AD cases at 96 weeks of age, was nine infants (PF versus SF, 95% CI, 4–182). 

#### 3.2.2. Respiratory Infections

**Figure 3:**
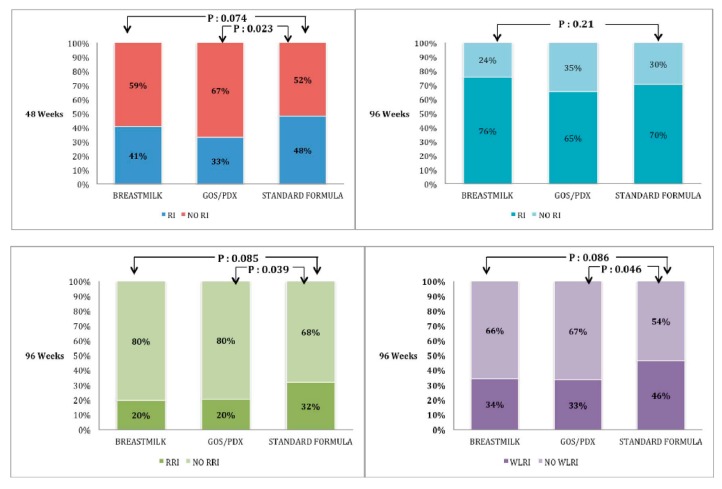
Percentage of infants with at least one episodes of respiratory infections (RI) at 48 and 96 weeks, with recurrent RI (RRI) until 96 weeks and with wheezy lower RI (WLRI) until 96 weeks of life.

Overall since two years old, 243 (70%) infants had at least one episode of RI (67% of FF and 76% of BF infants). As shown in Table 4 and Figure 3, both the number of infants with at least one episode of physician-diagnosed RI and the mean number of RIs episodes until 48 weeks were lower in PF infants than in SF infants (*p*: 0.023 and *p*: 0.007, respectively). These differences were no longer detected at 96 weeks after PF-containing formula withdrawal. The cumulative incidence of RIs was similar in PF infants and BF infants at 48 weeks and 96 weeks. The number of patients with RRIs until 96 weeks of life was lower in PF infants than in SF infants (*p*: 0.039). The number of infants needed to treat to prevent one case of recurrent RI until 96 weeks was nine (PF vs. SF, 95% CI, 5–193). The mean duration of RI episodes and parental workdays lost within 96 weeks were not different in the three groups. The number of antibiotic courses was not different in the PF and SF groups; however, a lower number of infants were prescribed antibiotics more than three times until 96 weeks of age in the former group (*p*: 0.05). Administration of more than three antibiotic courses within 96 weeks was significantly less frequent in BF infants than in SF infants (*p*: 0.004), but not in PF infants. Finally, the cumulative incidence of wheezing lower RI (WLRI) was lower in PF infants than in SF infants (*p*: 0.046) and similar to BF infants until 96 weeks. The number needed to treat to prevent one case of WLRI until 96 weeks was eight (PF versus SF, 95% CI, 2–129). All of the results about RI are reported in Table 4.

#### 3.2.3. Acute Gastroenteritis

Overall, since two years old, 177 (51%) infants had at least one episode of AG during the observation period (57% of FF and 41% of BF infants). The number of infants with at least one episode of physician-diagnosed AG until 36, 48 and 96 weeks was lower in the BF group and slightly but not significantly reduced in the PF group compared with the SF group (Table 5). No difference in the total number of diarrheal episodes and their mean duration of episodes among the three groups.

### 3.3. Intestinal Microbiology Substudy

#### 3.3.1. Intestinal Microbiology According to Type of Feeding

**Figure 4:**
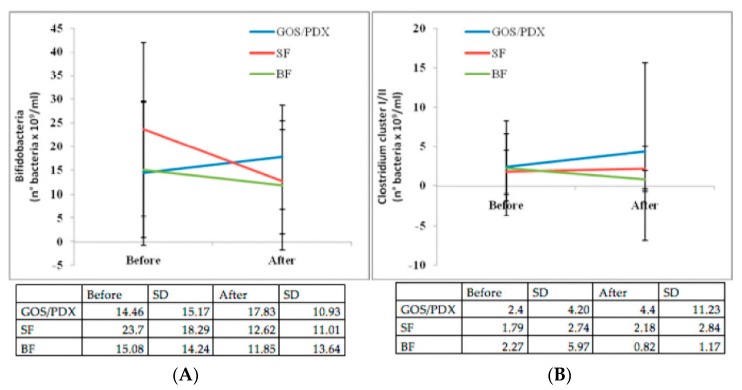
Bifidobacteria load (**A**, on the left) and Clostridium cluster I/II load (**B**, on the right) over time in the three nutritional groups (PF: prebiotic formula; SF: standard formula; BF: breastfeeding; SD: standard deviation).

Baseline Bifidobacteria loads increased after intervention in the PF group but not in the BF and SF groups. Bifidobacteria load after intervention was significantly greater in PF infants than either in BF infants (*p*: 0.007) or SF infants (*p*: 0.01) (Figure 4A). A progressive opposite trend in Clostridium cluster I load was observed in the three nutritional groups, with a progressive increase in PF infants, a decrease in BF infants and a stable colonization in SF infants (Figure 4B). The *F. prausnitzii* load was significantly higher in PF infants and SF infants than in BF infants (*p*: 0.05).

#### 3.3.2. Intestinal Microbiology According to the Development of Atopic Dermatitis and Common Infections

We analyzed the microbial composition of children with and without atopy at baseline and at 9–12 months. At post-intervention, AD-free infants had a significantly higher colonization with Clostridium cluster I (*p*: 0.02) than the AD group. This difference was not detected in the pre-intervention samples. There were no differences in the other bacterial species. Subsequently, the microbial populations between children with RIs (*n* = 99) and those without RIs (*n* = 48) were analyzed. Infants who were free from RIs had significantly increased load of Bifidobacteria compared with those without RIs (*p* < 0.001) in fecal samples collected at 9–12 months of life (after nutritional intervention), and this difference was not detected in the pre-intervention samples. No differences in other bacterial species were noted. Finally, microbial species were comparatively analyzed in infants who had no acute gastroenteritis until 96 months (*n* = 67) and those who had at least one episode of acute gastroenteritis (*n* = 80). No differences were found at baseline evaluations, whereas a lower Firmicutes/Bacteroides ratio was noted in infants without a history of acute gastroenteritis than in infants who had at least one episode of diarrhea (*p*: 0.013) at post-intervention evaluation. The obtained results provided a pattern driving nutritional intervention to clinical outcomes through bacterial species modifications.

## 4. Discussion

This is a double blind randomised clinical trial including also a parallel comparison group of BF infants. As for atopic dermatitis, which represents the primary objective, our study does not show a significant reduction of incidence of its occurrence in the PF group (configuring itself as a negative study). However it is important to underline that the risk of AD in PF compared with SF is reduced by 35%. A major clinical effect observed in PF-fed infants was the reduction of RIs. This effect may be important in at-risk population of infants born to atopic parents considering the vicious circle between atopy and infections. Children at risk of atopy have a greater risk of RIs [22], and in turn recurrent RIs trigger asthma [23]. In particular, WLRIs, such as bronchiolitis, are significantly associated with asthma later in life. In our cohort of infants, approximately 25% were free from RIs until 96 months of life, which agrees with that reported in the general population [5]. However, we observed a high rate of WLRIs with about 40% of infants presenting at least one such episode, with higher incidence than that expected in the general population [5]. WLRIs, representing the bridge between RIs and asthma [24], were highly prevalent in our cohort probably due to the atopic risk of infant and/or close physician surveillance. The effect of GOS/PDX on RIs was dependent on the assumed time since it was not observed when the enriched formula was discontinued. However, even this transient effect had a prolonged impact since the prevalence of RRIs and WLRI at 96 weeks was reduced in the PF group compared with the SF group. As for acute gastroenteritis, we obtained further proof of the well-known protective effect of BF. The rate of AG was not different in the PF and SF groups.

The mechanisms of the protective effect of prebiotic against RIs are not clearly elucidated but are likely to involve modifications of intestinal microbiota. In this study, we explored the modifications in intestinal microbiota in parallel with the clinical effects and confirmed that prebiotics increase the load of Bifidobacteria. The role of intestinal microbiota and RIs has already been established in chronic conditions, such as cystic fibrosis and asthma. In a previous study involving children with cystic fibrosis (CF), we found a disrupted intestinal microbiota with reduced diversity in intestinal microbiota composition and Bifidobacterial load was strongly reduced compared with non-CF children [8]. Our study directly showed for the first time that Bifidobacteria were increased in children without RIs regardless of nutrition, supporting the protective role of Bifidobacteria against RIs. More specifically, our data support the relationship between intestinal microbiota and direct protection from RIs involving Bifidobacteria. Therefore, the well-known “bifidogenic effect” of prebiotics has a clinical implication resulting in protection against RIs. The reduction of repeated antibiotic courses may well contribute to maintaining eubiosys in children receiving prebiotic-enriched formula.

Previous studies showed a positive association between Clostridia and atopic sensitisation [25,26], but the opposite [20,27] or no association was also reported [28,29]. In our study, Clostridium cluster I (also known as *Clostridium butyricum*) load was increased in AD-free infants after the intervention period but not at baseline. This pattern is similar to that reported by Nakayama et al. [27], but in contrast to that by Penders et al. [20]. In our study, Clostridium cluster I load was specifically increased in prebiotics but not by other nutritional groups (including BF). Interestingly, non-toxigenic Clostridium I strains are currently used as probiotics in Asian countries [29] in combination with immunotherapy in asthmatic patients [30] in order to alleviate intestinal inflammation in ulcerative colitis in food allergy [31] and to treat intestinal dysbacteriosis in neonates [32].

The present study is singular in this field from a methodological point of view, in particular for the following aspects: the accuracy in defining the standardised endpoints; the use of expert, fixed and blinded clinicians for the follow-up of infants; the use of stringent inclusion and exclusion criteria (such as the high risk of atopy of the studied population); the high statistical power of the study set to emphasize differences of 20%, which is more than considered in other studies, with nutritional intervention; and the presence of parallel clinical and microbiological data. Limitations of this study are mainly: the small size of the final population; the non-randomised BF control group; and the high percentage of mixed-fed (BF plus FF) infants, according to common infants feeding habits.

## 5. Conclusions

Infants fed a GOS/PDX-enriched formula demonstrated a more similar clinical pattern of outcome parameters to infants fed breast milk, but outcome parameters that were different from infants fed SF. In our study, we hypothesised a 20% reduction of major clinical outcomes, including atopy and respiratory infections. In a previous study, a reduction of less than 10% in the prevalence of atopy was observed [33], which is in line with our results. The reduced risk observed in RIs is statistically important and supported by a compelling state. In addition, we found a somewhat specific relation between PF-induced bifidobacterial load increase and protection from RIs and Clostridium cluster I, as well as protection from atopy. All of the data were obtained in a large population of infants at risk of atopy in a rigorously controlled trial. The findings in this study should be interpreted cautiously as they are far from being conclusive. Longer-term effects derived from the prevention of atopy and respiratory infections at early life and from early changes in microbiota structure need to be evaluated.

## Figures and Tables

**Table 1:** Baseline demographic characteristics of the infants per study group.

	PF	SF	BF	TOTAL	*P*
	(*n* = 118)	(*n* = 104)	(*n* = 123)	(*n* = 345)	
Delivery					0.085
Natural: *n* (%)	74 (62.7%)	69 (66.3%)	93 (75.6%)	236 (68.4%)	
Caesarean: *n* (%)	44 (37.3%)	35 (33.7%)	30 (24.4%)	109 (31.6%)	
Weight at birth (g)					0.33
Mean ± SD	3387.3 ± 378.3	3388.0 ± 424.1	3453.2 ± 370.7	3411.0 ± 390.2	
Median	3375	3352.5	3470	3390	
(25–75 percentiles)	(3209–3629)	(3036.2–3651.2)	(3195–3670)	(3170–3645)	
Length at birth (cm)					0.65
Mean ± SD	49.37 ± 1.72	49.57 ± 1.93	49.55 ± 1.81	49.49 ± 1.81	
Median (25–75 percentiles)	49 (48–51)	50 (48–51)	50 (48–51)	50 (48–51)	
Gestational age (weeks)					0.91
Mean ± SD	40.25 ± 2.29	40.34 ± 2.21	40.37 ± 2.06	40.32 ± 2.18	
Median (25–75 percentiles)	40 (39–42)	40 (39–41.7)	40 (39–42)	40 (39–42)	
First born: n (%)	56 (47.5%)	60 (57.7%)	58 (47.2%)	174 (50.4%)	0.21
Number of siblings	1 (0–1)	1 (0–1)	1 (0–1)	1 (0–1)	0.78
Median (25–75 percentiles)					
Mother’s age (years)					0.7
Mean ± SD	34.03 ± 4.53	34.35 ± 4.60	34.5 ± 3.96	34.29 ± 4.35	
Median (25–75 percentiles)	34 (32–37)	34 (31–38)	35 (31–38)	35 (31–37.5)	
Smoke in pregnancy: *n* (%)	5 (4.2%)	9 (8.7%)	5 (4.1%)	19 (5.5%)	0.24
Pets at home: *n* (%)	25 (21.2%)	35 (33.7%)	31 (25.2%)	91 (26.4%)	0.1
Mother’s education: *n* (%)					0.52
Lower secondary	9 (7.7%)	5 (4.8%)	5 (4.1%)	19 (5.5%)	
Upper secondary	52 (44.4%)	46 (44.2%)	47 (38.5%)	145 (42.3%)	
Degree	56 (47.9%)	53 (51.0%)	70 (57.4%)	179 (52.2%)	
Father’s education: *n* (%)					0.033 *
Lower secondary	23 (19.8%)	16 (15.4%)	8 (6.5%)	47 (13.7%)	
Upper secondary	53 (45.7%)	46 (44.2%)	57 (46.3%)	156 (45.5%)	
Degree	40 (34.5%)	42 (40.4%)	58 (47.2%)	140 (40.8%)	

PF: prebiotic formula; SF: standard formula; BF: breastfeeding; SD: Standard Deviation; n: number. * Statistically significant.

**Table 2:** Atopic dermatitis (AD) parameters in the three nutritional groups.

	PF	SF	BF	*P*
	*n* = 118	*n* = 104	*n* = 123	
AD episodes 36 weeks	44 (37%)	45 (43%)	48 (39%)	0.65
AD episodes 48 weeks	49 (41%)	50 (48%)	55 (45%)	0.62
AD episodes 96 weeks	56 (47%)	60 (58%)	61 (50%)	0.28
AD drug use 48 weeks *	33 (67%)	28 (56%)	31 (56%)	0.42
AD drug use 96 weeks *	39 (70%)	33(55%)	36 (59%)	0.25
AD drug use > 3 times (median) 48 weeks *	13 (33%)	13 (32%)	7 (13%)	0.025 ^(1)^
AD drug use > 3 times (median) 96 weeks *	16 (29%)	18 (30%)	11 (18%)	0.26
Mean number of AD episodes 96 weeks				
Mean ± SD	5.02 ± 8.44	5.56 ± 6.64	3.14 ± 2.87	0.11
Median (25–75 percentiles)	2 (1–5.25)	4 (1–7)	2 (1–4)	
Duration of AD (days)				
Mean ± SD	9.74 ± 7.44	8.39 ± 8.04	10.07 ± 8.83	0.51
Median (25–75 percentiles)	7.33 (4.5–12.5)	5.64 (3.25–11)	7.48 (4.42–14)	

PF: prebiotic formula; SF: standard formula; BF: breastfeeding; SD: standard deviation. * Percentages calculated on subjects with AD events; ^(1)^ PF vs. BF: *p* = 0.016; SF vs. BF: *p* = 0.020.

**Table 3:** Cox analysis to estimate AD risk considering potential covariates (hazard ratios and 95% CIs for the incidence of AD).

Variable	Hazard Ratio (95% CI)	*p*
Prebiotic formula (versus standard formula)	0.646 (0.369–1.129)	0.09
Breastfeeding (versus standard formula)	0.512 (0.309–0.850)	0.01 *
Breastifeeding (versus prebiotic formula)	1.02 (0.543-1.214)	0.71
Female sex (vs. male)	1.052 (0.684–1.619)	0.82
Pets at home (vs. no pets)	0.947 (0.575–1.561)	0.83
Postsecondary education of father	1.452 (0.922–2.286)	0.11
(vs. secondary)		
Smoke in pregnancy	0.84 (0.71–1.561)	0.92
Age of introduction of solid foods	1.422 (1.034–1.957)	0.03 *
No. of sibilings in household	0.895 (0.717–1.118)	0.33

**Table 4:** Respiratory infections (RI) parameters and incidence of wheezy lower RI among the three groups.

	PF	SF	BF	*P*
	*n* = 118	*n* = 104	*n* = 123	
Infants with RI (at least one) until 48 weeks	39 (33%)	50 (48%)	50 (41%)	0.074 ^(1)^
Infants with RI (at least one) until 96 weeks	77 (65%)	73 (70%)	93 (76%)	0.21
Infants with RRI until 96 weeks	24 (20%)	33 (31%)	24 (20%)	0.085 ^(2)^
Antibiotic use 48 weeks *	38 (72%)	43 (72%)	57 (85%)	0.12
Antibiotic use 96 weeks *	58 (75%)	58 (79%)	75 (81%)	0.69
Antibiotic use > 3 times (median) 48 weeks *	8 (15%)	8 (13%)	8 (12%)	0.88
Antibiotic use > 3 times (median) 96 weeks *	22 (29%)	26 (36%)	14 (15%)	0.008 ^(3)^
Mean number of RI episodes 48 weeks				
Mean ± standard deviation	1.9 ± 1.49	2.93 ± 1.11	2.2 ± 1.7	0.013 ^(4)^
Median	1 (1–2)	2 (2–4)	1 (1–4)	
Mean number of RI episodes/96 weeks				0.06 ^(5)^
Mean ± SD	4.21 ± 1.89	4.93 ± 2.11	3.8 ± 2.16	
Median (25th–75th percentiles)	4 (2–6)	4 (2–6)	3 (2–5)	
Duration of RI (days)				
Mean ± SD	5.85 ± 2.33	5.90 ± 2.68	5.99 ± 2.29	0.93
Median (25th–75th percentiles)	5.67 (4–7)	5.5 (4.32–7)	6 (4.9–7)	
Wheezy lower RI 48 weeks	33 (28%)	38 (36%)	36 (29%)	0.34
Wheezy lower RI 96 weeks	39 (33%)	48 (46%)	42 (34%)	0.086 ^(6)^
Number of parent workdays lost within 96 weeks	36 (30%)	28 (27%)	26 (21%)	0.25
Number of days lost	7.03 ± 7.56	6.57 ± 10.21	9.85 ± 23.03	0.66
Mean ± SD	5 (2–9)	3 (1–9)	3 (2–5.25)	
Median (25th–75th percentiles)				

PF: prebiotic formula; SF: standard formula; BF: breastfeeding; SD: Standard Deviation. ^(1)^ PF vs. SF: *p* = 0.023; ^(2)^ PF vs. SF: *p* = 0.039; SF vs. BF: *p* = 0.061; ^(3)^ PF vs. SF: *p* = 0.056; SF vs. BF: *p* = 0.004; ^(4)^ PF vs. SF: 0.007; SF vs. BF: *p* = 0.012; ^(5)^ SF vs. BF 0.021; ^(6)^ PF vs. SF: *p* = 0.046; SF vs. BF: *p* = 0.065. Frequency and percentage, mean ± standard deviation, median (25th–75th percentiles). * Percentages calculated on subjects with RI events.

**Table 5:** Acute diarrhea in the three nutritional groups.

	PF	SF	BF	*P*
	*n* = 118	*n* = 104	*n* = 123	
Infants with acute diarrhea (at least once) until 48 weeks	50 (42%)	50 (48%)	33 (27%)	0.003 ^(1)^
Infants with acute diarrhea (at least once) until 96 weeks	62 (52%)	65 (62%)	50 (41%)	0.004 ^(2)^
Mean number of diarrhea episodes at 96 weeks				0.16
Mean ± SD	2.52 ± 3.04	2.66 ± 2.77	1.78 ± 1.28	
Median (25–75 percentiles)	2 (1–3)	2 (1–3)	1 (1–2)	

PF: prebiotic formula; SF: standard formula; BF: breastfeeding; SD: standard deviation. ^(1)^ PF vs. BF: *p* = 0.011; SF vs. BF: *p* = 0.001 ^(2)^ PF vs. BF: *p* = 0.064; SF vs. BF: *p* = 0.001.

## Abbreviations

Prebiotics in the Prevention of Atopy	PIPA
Respiratory infection	RI
Fructooligosaccharide	FOS
Galactooligosaccharide	GOS
Polidextrose	PDX
Atopic Dermatitis	AD
Prebiotic (GOS/PDX)-enriched formula	PF
Breastfeeding	BF
Human nondigestible Milk Oligosaccharides	HMO
Randomized Controlled Trial	RCT
CONsolidated Standards of Reporting Trials	CONSORT
Recurrent RIs	RRIs
SCORing Atopic Dermatitis	SCORAD
Intention-To-Treat analysis	ITT
Per Protocol	PP
Standard Deviation	SD
Wheezing Lower espiratory-tract Illnesses	WLRIs
Acute Gastroenteritis	AG
Cystic Fibrosis	CF
Standard Formula	SF
